# Health of England in 1856

**Published:** 1859-01

**Authors:** 


### THE HEALTH OF ENGLAND IN 1856.*

THE Registrar-General's reports and returns always rank amongst the first literary sanitary papers published in this country. They are social as well as sanitary histories; and once on a time, before the penny-wise-and-pound-foolish principle entered into the hearts of Treasury Lords, these important state papers were at the command of every man who was anxious to know of the health and physical wealth of his fellow countrymen. A dogged determination, on the part of the Lords publishers, to carry on their little trade, has prevented many readers from the benefit of their ordinary useful readings. Medical men have felt the evils of the Treasury publishing scheme greatly. They, as a body, are not wealthy. They, as a body, contribute all the facts out of which the death-rate and health-rate of England are composed. This duty, costing both time and labour, was formerly rewarded by permission to read the result; more was never asked. That privilege cut off now peremptorily and without reason, many of those who read our pages apply to us to give them, as far as we are able, a condensed account of the last Registrar-General's annual doings. In the sequel, we endeavour to supply this demand.

Never has such a favourable report been presented as that for 1856. 657,453 children were born alive. This number is 22,410 in excess of the preceding year. Of these children,

* The Registrar-General's Annual Report for the Year 1856. London: Published by the Lords Commissioners of the Treasury, and sold by all Parliamentary Booksellers.

335,541 were boys, and 321,912 were girls; so that to every 100 girls, 104 boys were born alive. This proportion varies in different counties: thus in Wilts, 109 boys were born, to 100 girls; in Bucks, only 98 boys were born, to 100 girls. The natural increase in the population by excess of births over deaths was 729 daily, or 266,947 in the year; and even this number is too small, for, as no one is bound under penalty to give the registrar notice of a birth, many children are never entered on the register.

The birth-rate for the year was 3·452 per cent.; it is the highest on the English records.

*Marriages.* The number of marriages was unusually great; a sure sign of the prosperity of the country. 318,674 persons entered the matrimonial state, thereby founding 159,337 new families. This number is 7,224 greater than that for the preceding year. Early marriages are becoming much more frequent. Ten years ago, 13 in 100 of the young women who married were under age; now the proportion is 18 in 100.

The marriage register shews the pleasing fact, that an improvement in the elementary education of the people at large is going on. 71 per cent. of the men who were married wrote their names, and 29 per cent. made marks. Of the women, 60 in 100 wrote, and 40 made marks. Hence 13 in every 20 of the persons marrying could write. The deficiency is greatest on the part of the females; yet they have made much greater progress during the last fifteen years than the men. In 1841, only 51 per cent. of the women, and 67 per cent. of the men, who married could write.

*Deaths.* "390,506 deaths were registered in the year, or less by 35,197 than the deaths in the previous year; and the rate of mortality was 2·050, or little more than 20 deaths in 1000. This is the lowest rate observed. The average rate of mortality for the last nineteen years was 2·236, or 22 in 1000. . . . In some counties the mortality was unusually low: thus, the mortality was 15 in 1000 in Westmoreland; less than 16 in Dorsetshire and Lincoln. . . . The mortality of London was less than 22 in 1000. The mortality was greatest in Lancashire (24·54) and Durham (25·54); where, with all their energy, many of the governing bodies have left the large towns deplorably destitute of satisfactory sanitary arrangements."

*Ages at which death occurs.* "The mortality varies at every age. It is lowest at some period between the age of 10 and 15 years. In the year 1856, the rate at that age (10-15) was ·45 per cent. in both sexes, or 4·5 deaths in the year to 1000 living. The vital force, or the power of resisting dissolution, increases rapidly from the date of birth up to the age of puberty; so that every year a child's chances of living grow greater. After the age of puberty, when the body in every other respect is acquiring strength, the power of resisting disease declines slowly up to some age about fifty-five years, and then more rapidly. Thus, in the first five years of life, 68 in 1000 boys, and 59 in 1000 girls, die annually. In the second five years (5-10) of life, the proportion is 7 in 1000 in both sexes. In the ages commencing at fifteen, the deaths to 1000 males living in each of the successive periods of ten years, up to the age of fifty-five years, were nearly 7, 9, 12, 17; at fifty-five and upwards, 30, 61, 129, 263, and 322. The female mortality follows the same law; but it was lower at all ages, except 5-10, 15-35, 95 and upwards, than the mortality of males. At all ages, and in both sexes, the mortality was below the average in 1856."

Difference in temperature appears to have been a great cause of the difference in the mortality of England. In 1855, the average temperature of the first quarter was 34·1° Fahr.; of the corresponding quarter of 1856, the average temperature was 40° Fahr. The deaths in these two quarters were respectively 134,542, and 103,014; thereby shewing an excess of 31,528 deaths to be due to the severely cold winter of 1855.

Density of population also affects the death-rate of a district. Nearly one-half (over eight millions) of the population of England is lodged in large towns; and, while 25 in 1000 die in the town districts, the proportion is only 19 in 1000 in the country generally. The insalubrity of the dense districts arises not wholly from the fact of there being a large number of people congregated together, but in a great degree from the want of adequate means to redistribute over the earth the organic matter reduced to refuse in these large towns. Sanitary measures have greatly improved the health of thickly populated districts; but reform in sanitary matters, as well as in other cases, makes slow progress. Still density of population has much to do with the rate of mortality.

"In three districts of England, the annual mortality during ten years was at the rate of 15 deaths in every 1000 living; in fourteen districts, at the rate of 16; in forty-seven districts, at the rate of 17 in 1000 living. Where the mortality was 15, 16, and 17, the average number of persons living on a hundred acres was respectively 9, 17, and 22. Again, the annual mortality in the same period, in thirteen districts, was at the rate of 27 deaths to 1000 living; while in eighteen districts, the rates ranged from 28 to 36 deaths to 1000 living. The numbers of persons on a hundred acres in these districts were respectively 279 and 693."

There is a very marked difference in the mortalities of town and country districts in the summer quarter: in the former, 27 persons died to 1000 living; in the latter, the proportion was only 18 in 1000. This speaks well for the country ; but even here there is room for immense improvement in sanitary matters. The germs of insalubrity are scattered about in every village ; for the rational laws of health are violated alike in the cottage and in the farm-house. Dwelling houses are frequently built on the most unhealthy sites ; while the farmer surrounds his house with ricks, buildings, and trees, and, what is worse, the refuse of the house and of all the animals is kept month after month undergoing fermentation, and giving off noxious vapours. Can we wonder, then, that the cattle perish (unaccountably to the farmer), and that disease and death visit such abodes as these? In many minds, the English farmer is the personification of sound robust health ; but the Registrar-General's figures say, 6,426 farmers die in a year ; about three-fifths of them are under sixty-five years of age, and many of them are young men.

If then the country, for which nature does so much, demand improvements, how much more necessary is it that all our sanitary resources should be called forth to counteract the evil effects arising from the condensation of eight millions and a half of people in our large towns. "We want pure air and pure water about our dwellings, and the refuse, which infects the air and makes poison to man, to be restored directly to the soil." Attention to these matters, would render the population of our great country healthier and happier than it has ever been ; and scarlatina, diarrhœa, typhus, whooping-cough, and small-pox, which have made many a home desolate in this otherwise healthy year, would be far less frequent. England is already the healthiest country of Europe ; what position will it take when popular prejudice succumbs to sound reason, and when parish boards shall throw aside their party quarrels in consideration of the public weal.

390,506 persons died in England and Wales in 1856. Of these, about 324,000 died from diseases and accidents, which were, perhaps, inevitable ; but 67,000 persons died from causes which, if skilfully attacked, may be either mitigated, or removed altogether. Many of the 67,000 had lived to the ordinary limits of human life, but thousands in the throng had not lived out half their days. This is bad ; but 48,000 persons are now living who would have been dead, had the same rate of mortality prevailed in 1856 as in the ten preceding years ; and the loss of life from the causes just mentioned, is 35,197 less than it was in the previous year. Many of these lives have been saved by sanitary improvements.

Appended to the Report of the Registrar General is a letter from Dr. Farr on the causes of death, as far as they have been ascertained.

Of the 390·506 deaths in the year, the causes were not specified at all in 4,666 cases, and 3,474 are simply tabulated as sudden deaths, all inquiries having failed to elicit further definite information: 94,407 of the whole number of deaths were those of infants under one year old.

The causes of death are arranged in a few classes with numerous sub-divisions. 78,047 of the deaths are classified as zymotic diseases: typhus (15,398), scarlatina (14,160), and diarrhœa (13,815), proving fatal in 43,373 instances, or considerably more than one-half of the whole number due to this class. Hooping-cough, measles, and croup stand next in order. Small-pox, formerly so fatal, was the cause of death in 2,277 cases.

Of the class of diseases denominated "constitutional," 82,856 persons died. Phthisis or consumption stands sadly preeminent in this list, its victims numbering 48,950 persons, by far the greater proportion of whom were young women. "How many of the thousands of deaths are to be ascribed severally to the fatal stays and to the in-door life of women, etc. it is not easy to calculate. Air is the pabulum of life, and the effects of a tight cord round the neck, and of tight lacing round the waist, differ only in degree in the time of their manifestation and in some of their symptoms; for the strangulations are both fatal. To wear tight laced stays is, in many cases, to wither, to waste, and to die, and is perhaps the natural chastisement of the folly which inflicts this Chinese deformity, natural only to wasps and other insects, on the human figure." The tubercular diseases carried off in all 63,832 persons.

The "local diseases," as inflammations, the allied pathological phenomena or their results, and functional diseases of particular organs, proved fatal to 149,911 persons. 50,535 of these died of diseases of the brain and nervous system, including also 23,946 deaths by convulsions. 13,672 deaths were referred to diseases of the organs of circulation. Diseases of the respiratory organs proved fatal to 52,908 persons, 21,528 of whom died from bronchitis and 22,653 by pneumonia. These diseases, and all others of the class, were less fatal than in the previous year. 22,620 persons died from diseases of the digestive organs. The other causes of death under this heading are numerous, but the cases are few in each.

It is gratifying to observe the decrease of mortality in childbirth. The birth of 10,000 living children involved the deaths of sixty mothers in 1847, but the proportion of deaths has gradually decreased each year, until in 1856 it was reduced to forty-four. The lives of sixteen mothers were thus saved in every 10,000 children born.

The cases classified as premature birth and debility amounts to 17,997. Atrophy or wasting away was returned as the cause of death in 13,712 cases. 23,931 persons died of old age. 14,912 deaths arose from external causes, and therefore belong to the violent order of unnatural deaths. 237 deaths are referred to intemperance, 451 to delirium tremens. The mortality under both these heads has decreased during the last three years. Burns and scalds caused the deaths of 2,919 persons. 432 persons were poisoned, this number being 52 in excess of the previous year. 1,314 persons died from hanging and strangulation, and 2,681 from drowning. Carelessness in the management of mechanical forces is manifested in the deaths of 5,433 persons from fractures and contusions. Happily the deaths from these causes are gradually decreasing.

Few diseases are equally fatal to both sexes. Hooping-cough, influenza, dropsy, heart-disease, and consumption are more fatal to females than to males; but small-pox, diseases of the respiratory organs, and indeed almost all other diseases, are more fatal in their effects upon men than upon women.

It is remarkable that so many as 21,801 persons should have died in such a manner as to require a coroner's inquest. The verdicts were—murder 205, manslaughter 271, justifiable homicide 6, suicide 1,314, accidental deaths 9,716, natural deaths 7,102, *found dead* 3,183. Persons have questioned the wisdom of holding so many inquests, seeing that so few persons were ultimately convicted. It must, however, be remembered that 1,314 suicides are included in the lists, and after all the information that could be elicited, the verdict was, in 3,183 instances, *found dead.* No one surely will deny the importance of inquiry into such cases as these; nor must we measure the value of a coroner's inquest simply by crime found out. How many assassinations are prevented by fear of the after inquiry?

A few meteorological facts will conclude our review of this report for 1856. Greenwich is the point of observation.

The air in 1856 moved faster upon the average than it had done for the three preceding years. Its hourly rate was 4·244 miles, or nearly 4¼ miles, thereby giving an excess of velocity of about three miles per day. The rain fall was 21·9 inches, which is slightly below the average of the last eight years. The mean annual temperature was 49·1° Fahr., an average degree.

